# Integrating Niche Dimensions to Advance the Ecological Study of the Americas' Smallest Cat: The Guigna in Argentine Patagonia

**DOI:** 10.1002/ece3.73704

**Published:** 2026-05-30

**Authors:** M. M. Guerisoli, G. Bauer, S. Sarra Pistone, E. Buffa, C. Bonaglia, A. J. Giordano, M. I. Schiaffini

**Affiliations:** ^1^ Laboratorio de Investigaciones en Evolución y Biodiversidad (LIEB), CONICET‐Facultad de Ciencias Naturales y Ciencias de la Salud Universidad Nacional de la Patagonia San Juan Bosco Esquel Chubut Argentina; ^2^ Grupo de Investigación en Eco‐Fisiología de Fauna Silvestre (GIEFAS; INIBIOMA‐CONICET‐AUSMA‐UNCo) Neuquén Argentina; ^3^ S.P.E.C.I.E.S.—The Society for the Preservation of Endangered Carnivores and Their International Ecological Study Ventura California USA; ^4^ División Conservación y Manejo, Departamento de Conservación y Educación Ambiental Parque Nacional Los Alerces Villa Futalaufquen Chubut Argentina; ^5^ Facultad de Ciencias Exactas y Naturales Universidad Nacional de Cuyo Mendoza Argentina; ^6^ Dipartimento di Scienze Chimiche, Della Vita e Della Sostenibilità Ambientale Università di Parma Parma Italy; ^7^ Center for Human‐Carnivore Coexistence Colorado State University Fort Collins Colorado USA; ^8^ Center for Collaborative Conservation Colorado State University Fort Collins Colorado USA

**Keywords:** Felidae, habitat use, kodkod, Los Alerces National Park, temporal ecology

## Abstract

The guigna has a very restricted geographic range in southern South America, encompassing much of the temperate forests of Chile and Argentina. In the latter country, scientific information about its ecology is anecdotal. We present our findings from the first multi‐annual survey of guigna in Argentine Patagonia. We used 35 camera traps in a protected area with over 8000 camera‐trap nights, yielding a total of 176 independent events. We investigated habitat use through generalized mixed models and built predictors based on Grinnellian and Eltonian Niche variables. We also investigated the temporal activity patterns between years and temperature‐based seasons, comparing also the activity of melanic and spotted individuals. We found that habitat use for guignas was negatively affected by the presence of co‐predators and open vegetation; conversely, it was positively affected by the occurrence of thick forests and presence of potential prey. Guignas were mainly nocturnal, despite the relative lack of human impact; in contrast to some prior studies, we found no significant differences in activity of individuals with melanistic and spotted phenotypes. Notably, our study resulted in one of the highest detection rates recorded for the species anywhere, perhaps owing to either a greater availability of natural resources and/or the relative lack of anthropogenic impacts inside the protected area. Whereas the potential for intraguild predation or interference competition may have unsurprisingly had a negative impact on guigna presence, thick forests yielding greater prey availability, perhaps due to greater micro‐habitat heterogeneity, appeared to positively influence it. This important study is the first extended research study for the guigna in Argentina; in particular, it is the first to investigate the variables influencing seasonal and annual habitat use and to more extensively investigate diel and seasonal activity patterns, helping to identify crucial scientific limitations that should be further explored in the future.

## Graphical abstract

We present our findings from the first multi‐annual survey of guigna in Argentine Patagonia. We found that habitat use for guignas was negatively affected by the presence of co‐predators and open vegetation; conversely, it was positively affected by the occurrence of thick forests and the presence of potential prey. Guignas were mainly nocturnal, despite the relative lack of human impact; in contrast to some prior studies, we found no significant differences in the activity of individuals with melanistic and spotted phenotypes.

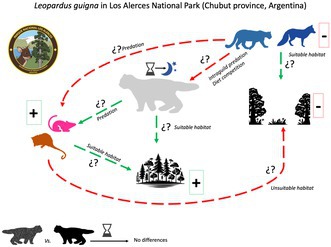

## 1 Introduction

Over the years, two main conceptual paradigms for the word “habitat” have repeatedly emerged: one that focuses on distinct vegetation categories existing in a given space, and another based on the definition of a “niche,” that is, one based on those resources and environmental conditions that allow for the survival and reproduction of wildlife populations (Gaillard et al. 2010). This second definition is rooted in niche theory, and suggests that habitats might vary according to the variables used and scale considered (Hutchinson 1957, 1978; Soberón 2007). Thus, at smaller spatial scales, variables relating to resources that interact with a given species (i.e., bionomic variables *sensu* Hutchinson 1978), exist as the *Eltonian niche* (Elton 1927). In contrast, at larger spatial scales where distributional limits are relevant, those variables that do not interact reciprocally with the studied species (i.e., scenopoetic variables *sensu* Hutchinson 1978), define the *Grinnellian niche* (Grinnell 1917). Using set theory, Soberón and Nakamura (2009) explained that different combinations of variables determine the components of a niche: an area accessible through dispersal (M), biotic variables (B), and abiotic variables (A). Assuming that everything about all A and B variables (*sensu* Soberón and Nakamura 2009) could be measured, then theoretically a niche could be fully understood (Hutchinson 1957). However, as only certain A and B variables can often feasibly be measured, habitat should be considered a portion of the Realized Niche, that is, an intersection of sets B, A, and M (see Soberón and Nakamura 2009).

A key point in understanding a niche is that the conditions that support a species' survival and reproduction are not necessarily the same for other species: that is, habitats are species‐specific (Krausman 1999). The habitat use of a species is important to study because it provides a way to understand how a species uses different resources for feeding, breeding, nesting, denning, and other life history traits (Krausman 1999). Such natural history traits are not necessarily performed in the same geographic locations, but sometimes across different habitats, or a habitat changing seasonally or within the same year (e.g., Dagtekin et al. 2024). Thus, time can be considered another dimension of the niche, as traits performed in the same habitat can vary temporarily, whether by day, season, weather, or on an inter‐annual basis (Schoener, 1974). Animals will often aim to maximize the timing of behaviors to increase their fitness (Hayward and Slotow 2009).

Knowledge about natural history traits for a given species is unequal across taxa, and even for the same taxa between adjacent regions (e.g., Kelt and Meserve 2014). For South American carnivorans, robust data on ecology is scarce (Carnivora; Prevosti and Pereira 2014), particularly for small felids (Felidae; Lucherini et al. 2004). Among them, the enigmatic guigna *Leopardus guigna* (Molina 1782) is one of the least known felid species in the western hemisphere. It has the most restricted geographic range of all New World felids, only encompassing a portion of the temperate forest of southern Chile and Argentina (Cuyckens et al. 2014; Napolitano et al. 2014; Zamora‐Cornejo et al. 2025). The guigna is a “Least Concern” (IUCN) species (Gálvez et al. 2025), threatened by habitat fragmentation, habitat loss, retaliatory killing for poultry predation, and the effects of climate change (Cuyckens et al. 2014; Napolitano et al. 2015; Gálvez et al. 2025). Habitat use and temporal activity patterns for this species are scarcely known, and most of the limited scientific information is based on studies from different habitats in Chile (see Dunstone et al. 2002; Zúñiga et al. 2009; Gálvez et al. 2013, 2017; Schüttler et al. 2017). In these regions, the guigna is depicted mainly as a forest dweller that also uses patches within agricultural areas (Gálvez et al. 2013, 2017; Schüttler et al. 2017). It has been proposed they can coexist with other sympatric carnivorans by segregating their habits and activity in time (Zúñiga et al. 2009).

Argentina represents only a minor portion of the geographic range of the guigna (Gálvez et al. 2025). To date, no investigation of its habitat use, activity, and seasonal temporal patterns has yet occurred in the country, making such research important to conservation planning (Monteverde et al. 2019). Actually, in Argentina, the guigna has only been recorded from four National Parks. Of them, Los Alerces National Park (LANP) is the closest one to its austral distribution limit (see Monteverde et al. 2019). To date, no detailed multiannual survey to investigate their use of habitat and diel activity patterns has occurred for the guigna in Argentina; rather, there have been some contributions related to interspecific interactions (Lucherini et al. 2001; Lucherini and Vidal 2003), conservation actions (Monteverde and D'Oliveira 2010), and the discovery of new sites where guignas occur (Berrondo and Bravo 2022; Agostini et al. 2024).

Our goal was to finally investigate habitat use in the guigna, and this small cat's activity patterns with respect to habitat. Using the Realized Niche (see established above) as the theoretical basis for the analysis of habitat use, we aimed to study the spatial and temporal dynamics, and habitat use through combinations of biotic and abiotic variables. We did this by conducting a multiannual camera‐trap survey in an Argentinean protected area in Patagonia, near the edge of this small threatened felid's range. The framework for this study is based on limited references to the guigna in the published literature, and studies on felid species where necessary (see Table 1).

**TABLE 1:** Hypotheses and predictions of the Niche Dimensions (i.e., habitat use and temporal activity) for guigna.

Niche dimensions	Hypothesis	Prediction	Variables
Habitat use	Sites with dense vegetation coverage have been associated with guigna's presence (Gálvez et al. 2013, 2017; Schüttler et al. 2017)	We expect guigna habitat use will be positively affected with forest cover and/or variables associated with dense vegetation	Map of vegetation coverage of the study area, Shannon Diversity Index, NDVI
	Small mammals represent the prey most frequently consumed by guigna, as in other parts of its distribution (Figueroa et al. 2018)	It is expected to have a positive relation between guigna's and prey's presence	Presence/absence of small mammals
	The presence of sympatric carnivorans can trigger shifts in the behavior and spatial ecology of guigna (Caro and Stoner 2003; Zúñiga et al. 2009; Havmøller et al., 2024).	We expect sympatric carnivorans to have a negative effect on guigna habitat use via their presence	Presence/absence of sympatric carnivorans
Activity pattern	The guigna is a nocturnal species largely because that is when their small mammal prey is active (Delibes‐Mateos et al. 2014; Gálvez et al. 2021; Zúñiga et al. 2025)	We expect a high degree of overlap between guigna and small mammal activity during the night hours	Independent guigna and small mammal record events
	Smaller carnivores often avoid larger sympatric carnivores through segregating their activity patterns (e.g., Krag et al. 2023; Havmøller, Wahyudi, et al. 2024; Havmøller, Parsons, et al. 2024)	We expect the guigna to temporally partition use of key areas where they are detected, in order to avoid other carnivorans and facilitate coexistence with them	Independent record events of guignas and other sympatric carnivorans (e.g., pumas, foxes)

*Note:* The variables we used are described in the Methods section.

## 2 Methods

### 2.1 Study Area

**FIGURE 1:**
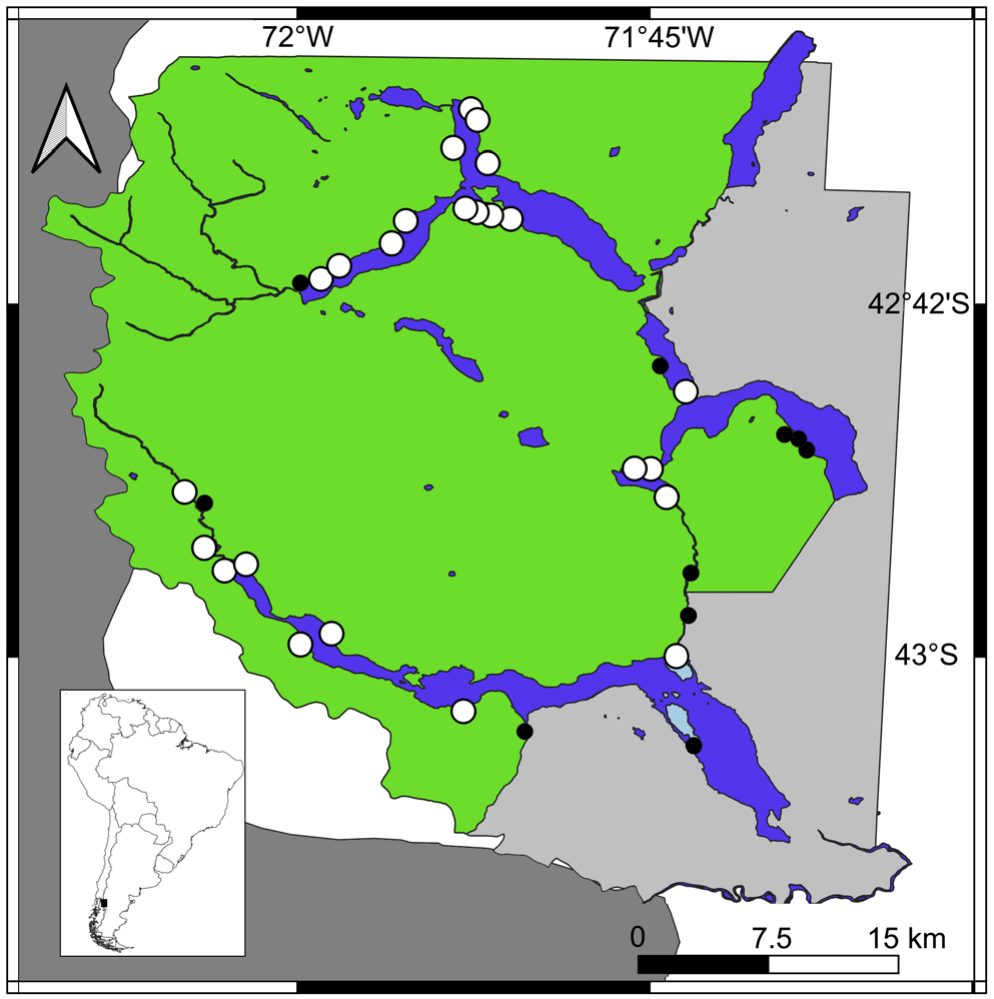
The location of LANP near the border between Argentina (white) and Chile (dark gray), and the park's two broad management units (Core National Park is in green, and National Reserve is shown in light gray). Camera trapping stations are represented by circles (white circles indicate presence of *L. guigna* , and black circles where no presence was indicated); the main water bodies are depicted in blue. The inlet is showing the location of LANP (black square) in South America.

Our study was carried out in LANP, a protected area (PA) in Chubut province in northwestern Argentine Patagonia (Figure 1). This PA occurs within the Valdivian Temperate Forests (*sensu* Dinerstein et al. 2017), a biodiversity hotspot with over 1500 endemic plant species threatened by habitat loss and fragmentation (Hora et al. 2022). This ecoregion occupies 346,000 km^2^ across Chile and Argentina from 36° to 47°S, and includes both complex and open temperate forests (Tecklin et al. 2011). The climate is generally cold, with a mean annual temperature of 8°C, and precipitation decreases abruptly from west to east along a gradient; more than 3000 mm/year of rain falls to the west of the Park, whereas roughly 800 mm/year falls further east, most of which occurs during the southern hemisphere winter (April to October; APN 2019). There are two main management categories within LANP (see Figure 1): (1) an inaccessible National Park *sensu stricto* of 188,379 ha (category II of Dudley 2008) which contains a vast network of rivers and lakes, free of human occupation and any roads; and (2) a National Reserve (category VI of Dudley 2008) containing 71,443 ha, encompassing rural human occupation together with roads, and also the main administrative services of the PA (APN 2019). This study holds all the necessary permits provided by Administración de Parques Nacionales (APN #1705).

### 2.2 Data Collection and Sampling Design

To detect the presence of guigna, we installed 35 camera traps (CTs) in LANP between 2021 and 2024 (Figure 1). The LANP is characterized by a limited accessibility by foot and/or vehicle, and remote areas (as those mainly sampled in this study) were reachable onyl by boat. Due to these challenges in gaining access to broad parts of the park (see CTs location in Figure 1), sampling locations were in part influenced by our ability to access those areas (see video in Supporting Information S1). All CTs (Bushnell TrophyCam HD, Bushnell, USA) were unbaited and set between 20 and 60 cm (Sereno‐Cadierno et al. 2026) above the ground to operate 24 h. Each CT was set to take three pictures in rapid succession upon activation, and set apart from the next closest CT by a minimum distance of 1 km; to minimize the potential for spatial autocorrelation of detection data among sampling units (O'Brien 2011). We chose this distance because it was a good compromise between the general lack of information for the species, and estimates of its home range from other nearby regions (i.e., 0.3–22.4 km^2^; Monteverde et al. 2019). Since the distribution of the guigna in this geographic area overlaps with the similar congeneric Geoffroy's cat *Leopardus geoffroyi* (see Monteverde et al. 2019; Pereira et al. 2019), guigna were positively identified using the information provided by Peckham (2023) and references therein.

We defined each independent event as no more than one picture of a species captured within the same 1‐h interval (e.g., Novoa et al. 2025). We defined total sampling effort as the product of the total effective days for the sampling operation, and all CTs in the study (i.e., camera‐trap nights). We expressed the capture rate for each species as the number of independent events divided by the total sampling effort, then multiplied by 100 trap‐nights. Considering that this felid presents both melanistic and spotted phenotypes (Schneider et al. 2015), we also calculated the number of independent detection events for both phenotypes.

### 2.3 Habitat Use

We fitted a Generalized Linear Model (GLM) with independent detection events of guigna as the response variable, and different variables pertaining to Grinnellian and Eltonian niches which could be affecting the spatial use of this felid as independent variables. These include (1) vegetation coverage, (2) Shannon Diversity Index, (3) the Normalized Difference Vegetation Index (NDVI), (4) the differential presence and absence of potential prey, and (5) presence of potential predators.

#### 2.3.1 Vegetation Coverage

Considering that sites with dense vegetation and canopy coverage (e.g., forest) have previously proven important to the presence of guigna (Gálvez et al. 2013, 2017; Schüttler et al. 2017), we used a map of vegetation coverage assembled by Mohr‐Bell et al. (2019). First, we created a 1 km buffer around each CT, and then calculated the proportions of different vegetation categories excluding areas represented by water. We implemented a 1 km buffer around each CTs to approximate the potential area used by guigna that could be detected at a given site. A 1 km radius corresponds to an area of, approximately, 3.14 km^2^, which falls within the range of home range sizes reported for the species in southern Chile (see above; e.g., Dunstone et al. 2002; Freer 2004).

Next, we classified the following vegetation categories: (i) Forested Land (“FL”; vegetation with > 7 m of height, coverage > 20%, and species composition > 80%), (ii) Mixed Forested Land (“MFL”; vegetation > 7 m in height, coverage of > 20%, and species composition < 80%), and (iii) Other Woody Formations (“OWF”; coverage resulting from major disturbances with coverage < 20%, see Mohr‐Bell et al. 2019). We followed Mohr‐Bell et al. (2019) in describing the “species composition” of each vegetation category. All geoprocessing was performed with QGIS (QGIS Development Team 2023).

#### 2.3.2 Shannon Diversity Index (SHI)

Since patch configuration can affect the use of space by guigna (Schüttler et al. 2017), we included the SHI to explore the relationship between patch diversity and habitat use for guigna in the study area. Again, we followed Mohr‐Bell et al. (2019) to define this coverage and created 1 km‐buffers around each sampling unit. This index presents as a null value (= 0) when there is a single patch in the buffer and increases in value as the number of different patches increases (Nagendra 2002). For our study, we used this index to assess patches through the QGIS (QGIS Development Team 2023) plug‐in GeoBioTool (Kim et al. 2025).

#### 2.3.3 NDVI

NDVI has been used to predict the spatial distribution of several different species of felids (e.g., Zuliani and Monjeau 2022; Araujo et al. 2023). We extracted NDVI values for each CT site using the tool TESViS in ORNL DAAC (2018) in QGIS. Because NDVI values are kept current and available every 14/16 days (see ORNL DAAC 2018), we extracted the information relevant to the corresponding time periods in which each camera trap was active. We therefore extracted the values corresponding to the first days of the months of February, May, August, and November, and averaged them for each camera trap site.

#### 2.3.4 Presence & Absence of Potential Prey

Because small mammals represent the most frequently consumed prey by guigna in other areas of its distribution (Figueroa et al. 2018), we recorded the “presence” of potential guigna prey through small mammal detection events of Rodentia: Sigmodontinae and *Dromiciops gliroides* (Figueroa et al. 2018) for each CT site (i.e., with “absence” as no detection of these species). Due to the low relative abundance of potential prey detections across our extensive sampling effort, we prefer here to use the variable as a dichotomous one. Species identification was not possible due often to blurry images, and/or a lack of diagnostic characters visible in camera‐trap pictures (e.g., fur color, dental characteristics; Pearson 1995).

#### 2.3.5 Presence and Absence of Co‐Predators

Since the presence of sympatric carnivorans can trigger shifts in behavior and spatial ecology of another species (Caro and Stoner 2003; Havmøller et al., 2024), we included other co‐occurring carnivorans as predictor variables potentially affecting guignas. For our study, we defined the “presence” of co‐predators as any event that included a puma *Puma concolor* and/or Andean fox *Lycalopex culpaeus* at the CT site; we concluded these species were “absent” when they were not detected. Although there were other co‐occurring carnivorans in our study area, including Geoffroy's cat, hog‐nosed skunk *Conepatus chinga*, little grison *Galictis cuja*, huillin *Lontra provocax*, and the introduced American mink *Neogale vison*, we did not include them given their low frequency or complete lack of detection events.

### 2.4 Data Processing and Ecological Modeling

To assess for potential collinearity among quantitative variables (i.e., “FL”, “MFL”, “OWF”, “SHI”, “NDVI”), we conducted an autocorrelation analysis in R (R Core Team 2024). If correlation coefficient values (r) were ≥ 0.7, we considered these variables to be “highly correlated” (Dormann et al. 2013). All numeric variables were scaled and the GLM was fitted with a negative binomial distribution (Zuur et al. 2009; Lindén and Mäntyniemi 2011). As an offset of the GLM, we also used sampling effort per camera trap. We analyzed independence between sampling sites (i.e., presence/absence of spatial autocorrelation) by checking the Moran's I (Moran 1950) in the residuals of the best model using a Moran Test with package “spdep” (Bivand and Wong 2018). We ultimately found there to be a lack of spatial autocorrelation (Moran's *I* = 0.077, *p* = 0.132), which indicated spatial independence in the placement of camera traps. We performed a multi‐model inference and selection framework using the function “dredge” in MuMin package (Bartoń 2025) selecting the model with the lowest AIC (Akaike Information Criteria; Akaike, 1974; Burnham and Anderson, 2004). Multicollinearity between variables was evaluated through the Variance Inflation Factor (VIF). Residuals were analyzed using the “simulateResiduals” function in DHARMa package (Hartig 2016). These analyses were performed using the R packages “Car” (Fox et al. 2012), “lm4” (Bates et al. 2015), “MuMin” (Bartoń 2025), and “MASS” (Venables and Ripley 2002).

### 2.5 Activity Patterns

To understand activity patterns, we extracted the date and time data for each of the guigna independent detection events. Those pictures which had date and time listed incorrectly due to CT malfunction were not considered for activity pattern analyses. We examined the activity patterns of guigna throughout the year, and compared and contrasted the colder (21 March–20 September) and warmer (21 September–20 March) seasons. We also considered the day phase (i.e., night, sunset, day, sunrise) through the online tool (https://www.timeanddate.com/), by averaging the time of sunrise and sunset through the year, as well as by cold and warm seasons (e.g., Guerisoli et al. 2019). Sunrise and sunset phases were created by adding 1 h from and to sunrise and sunset times (i.e., phases of 2 h each; e.g., Guerisoli et al. 2019). We then applied the Jacob's Index of Selection (JIS) to the day phases to understand if there was a preference for any phase (i.e., throughout the year, cold, warm seasons; Jacobs 1974, e.g., Guerisoli et al. 2019), and characterized the activity patterns of guigna with Kernel density plots (i.e., throughout the year, overlap for cold vs. warm seasons; Ridout and Linkie 2009). In addition, we characterized the activity of melanistic and spotted phenotypes separately using the JIS, and assessed for overlap in independent events for both phenotypes, using Kernel density plots (Meredith and Ridout 2016). To understand how the guigna's activity pattern could be affected by co‐predators and small mammals (i.e., potential prey; see above), we looked for overlap in the guigna Kernel density plots with those of pumas, foxes, and potential prey. For all potential overlap in activity, we calculated the activity overlap coefficient (Dhat1) and its CI (Meredith and Ridout 2016), and followed Monterroso et al. (2014) to examine the ranges for the data we collected. As such, Dhat1 score ≤ 50th percentile was considered a “low activity overlap value”, 50th percentile < Dhat1 ≤ 75th percentile was considered a “moderate overlap value”, and Dhat1 > 75th was considered a “high overlap value” (Monterroso et al. 2014). We then used the Watson's *U* ^2^ statistic (*⍺* = 0.05) to evaluate for significant differences between the Kernel curves (Jammalamadaka and SenGupta 2001). Thus, when *U* ^2^ > “critical value,” we concluded significant differences existed (i.e., the data come from different statistical distributions; Jammalamadaka and SenGupta 2001); in contrast, when *U* ^2^ < “critical value,” we concluded there were no significant differences (i.e., data came from the same statistical distributions; Jammalamadaka and SenGupta 2001). We tested the data with a Herman‐Rasson's test of uniformity (Landler et al. 2019; Havmøller et al., 2024). All analyses were performed using the R‐packages “circular”, “overlap” and “CircMLE” (Meredith and Ridout 2016; Fitak and Johnsen 2017; Landler et al. 2019; R Core Team 2024).

## 3 Results

We logged a total of 176 independent guigna detection events with a sampling effort of 8024 night‐traps (2.19 independent events/100‐night traps). Of these, 37.5% (*n* = 66) of these events were of the melanistic phenotype.

### 3.1 Habitat Use

**FIGURE 2:**
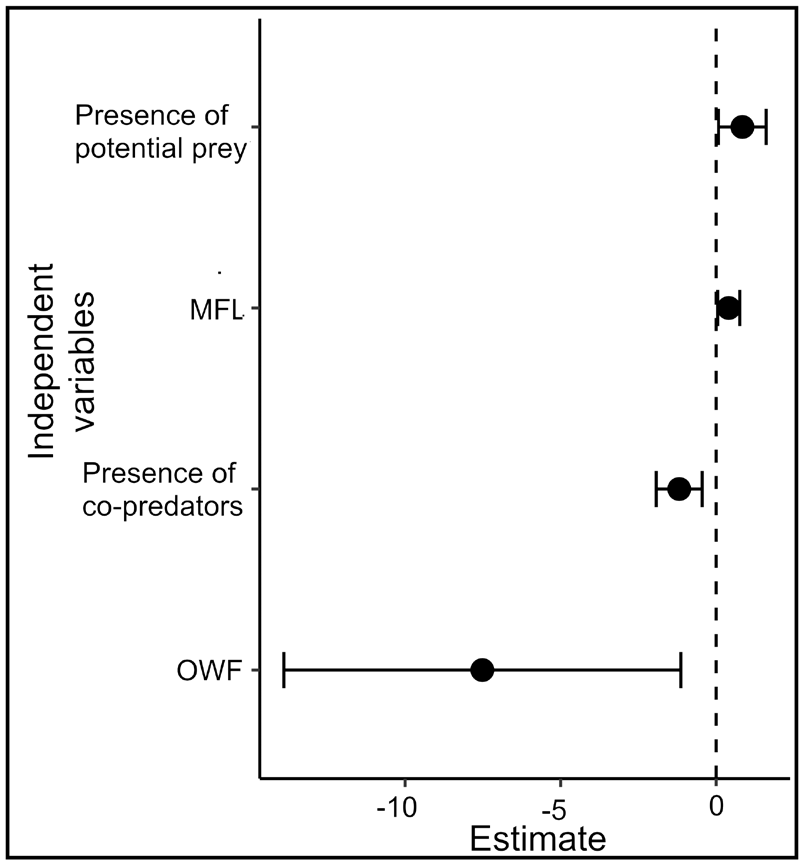
Estimates of the top model with the lowest AIC (i.e., 151.53). Confident intervals for the independent variable's estimates. “MFL”: Mixed Forested Land; “OWF”: Other Woody Formations.

Because we found two categories on our vegetation map to be correlated (i.e., *r* = −0.702: “FL” vs. “MFL”), we ran the GLM only with: “NDVI”, “MFL”, “OWF”, “SHI”, and the presence/absence of potential prey and co‐predators; we therefore eliminated “FL”. A check for multicollinearity revealed VIF values of: 1.65 for the presence/absence of co‐predators; 1.30, for “SHI”; 1.04 for the presence/absence of potential prey; 1.72 for “NDVI”; 1.16 for “MFL”; 1.21 for “OWF”. These results indicated no multicollinearity between these variables. Residual diagnostics indicated no significant deviation from the expected distribution (KS test *p* = 0.34), no evidence of overdispersion (*p* = 0.2), and no outliers (Supporting Information S2). However, the residuals versus predicted values plot indicated a slight quantile deviation, suggesting some structure not fully captured by the model (Supporting Information S2). Top model (AIC = 151.53) reported that “presence of co‐predators” and higher proportions of “OWF” negatively affected guigna occurrence, whereas both the “presence of potential prey” and higher proportions of “MFL” both affected guigna positively (Figure 2; Supporting Information S3).

### 3.2 Activity Patterns

**FIGURE 3:**
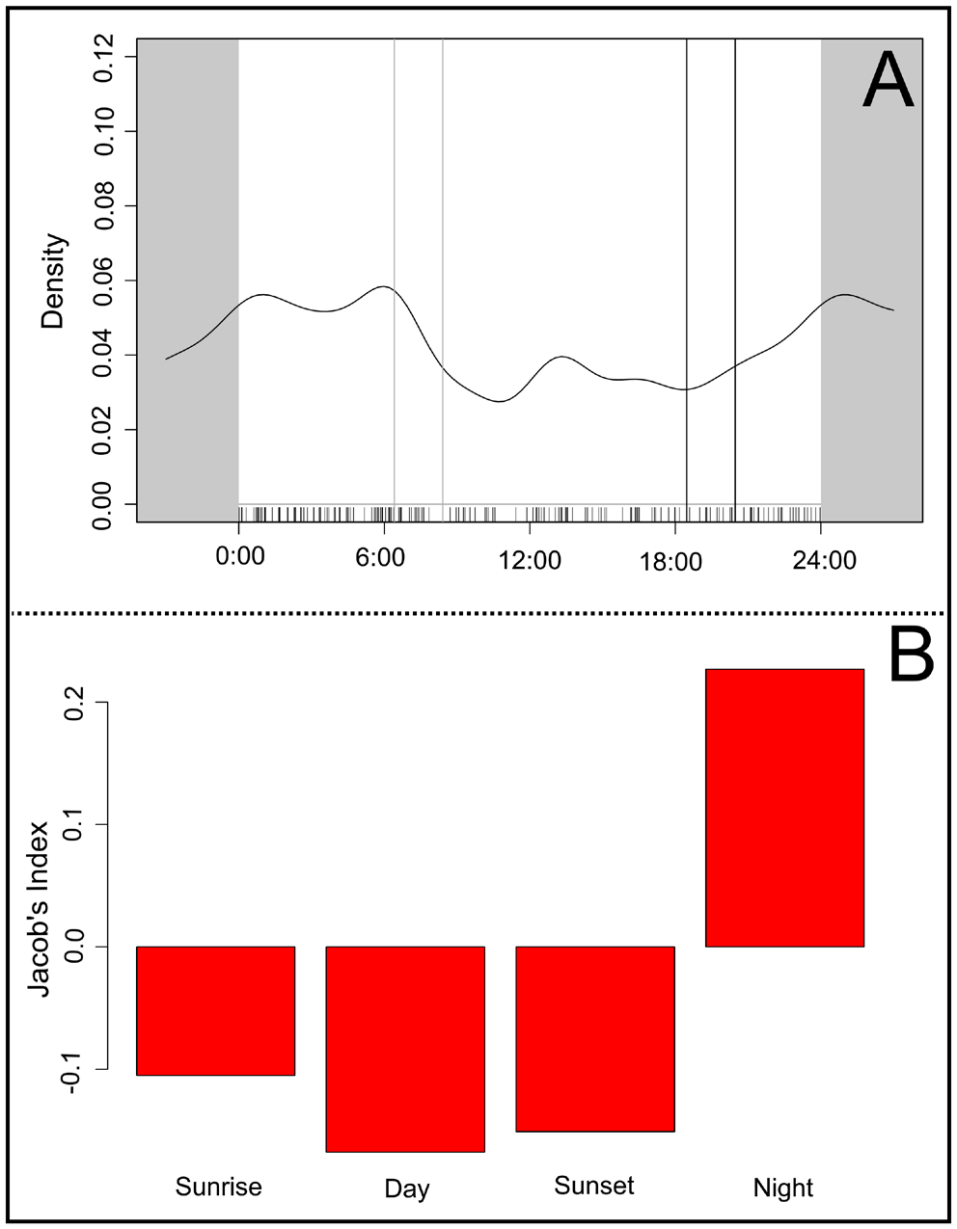
(A) Guigna activity patterns throughout the year: Gray and dark lines indicate sunrise and sunset phases, respectively. (B) Jacob's index selection for guigna activity pattern throughout the year.

Most independent guigna detection events, *n* = 175 (i.e., events with correct data and hour) occurred during the night phase, with their frequency increasing after sunset (*T* = 7.22, *p* = 0.036; Figure 3A). Based on these events therefore, we saw a selection for the night phase (JIS = 0.22), and a slight avoidance for all other phases (Figure 3B).

**FIGURE 4:**
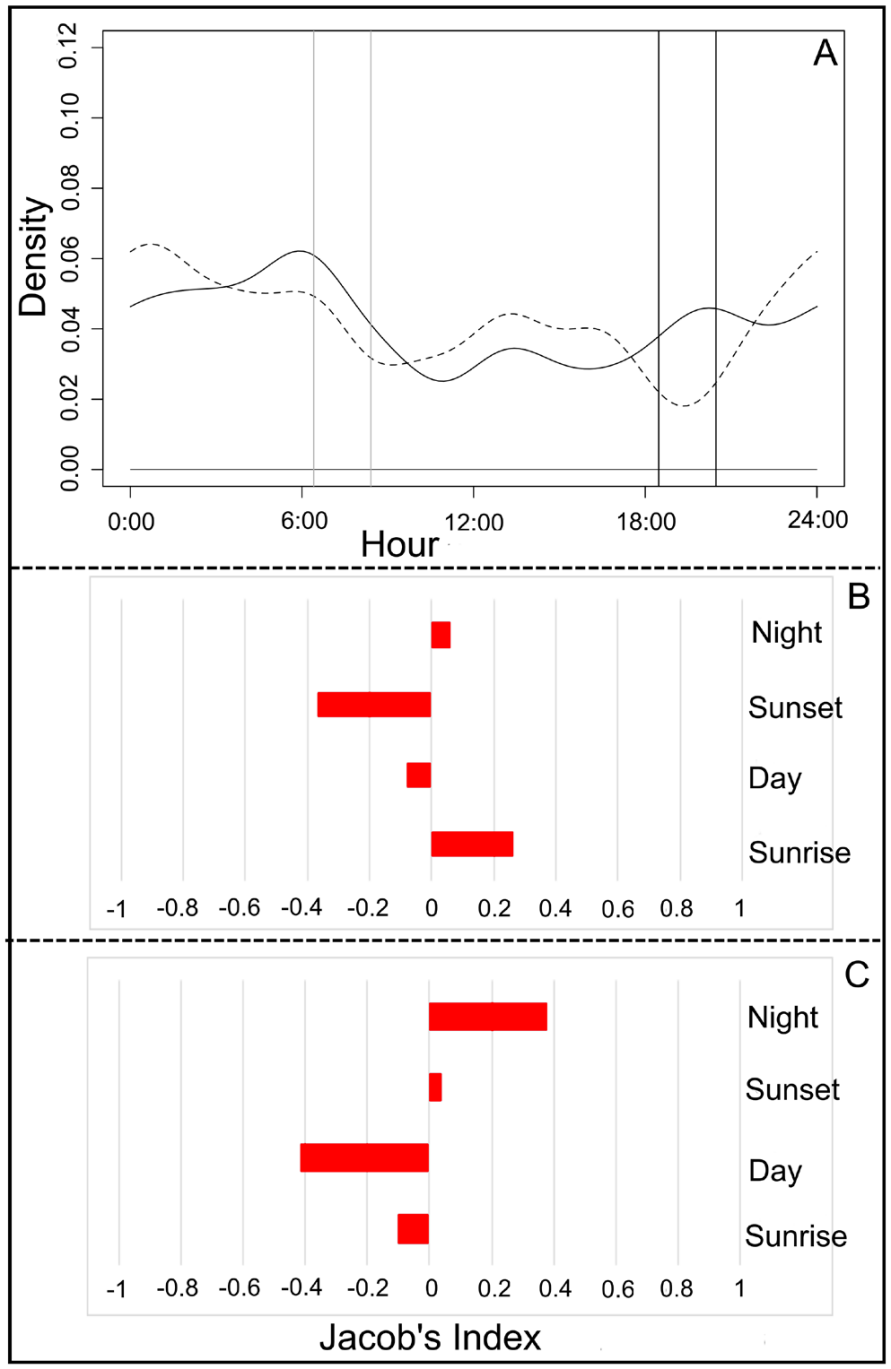
(A) Overlap of cold (continuous line) and warm (dashed line) seasons guigna activity pattern. Gray and dark lines indicate sunrise and sunset phases, respectively. Jacob's index selection for guigna activity pattern during the warm season (B) and during the cold season (C).

During the warmer season, guigna detection events (*n* = 75) mainly occurred during the night and day phase. Interestingly during this season, crepuscular activity, that is, the frequency of detection events occurring around sunrise and sunset phases, declined overall relative to the colder season (*T* = 5.33, *p* = 0.13; Figure 4A). Despite this reduction in crepuscular activity overall, guigna activity patterns still suggested a preference for the time around sunrise (JIS = 0.26), and a slight avoidance of the time around sunset (JIS = −0.36; Figure 4B). During the colder season, detection events (*n* = 100) occurred mostly at night, whereas these detections decreased after sunrise and increased in frequency after sunset (*T* = 5.09, *p* = 0.15; Figure 4A). During the cold season, guignas appeared to exhibit more nocturnal activity (JIS = 0.38) and to altogether avoid being active during the day (JIS = −0.41; Figure 4C). However, overall we found the overlap in Kernel activity density curves between cold and warm seasons was high, and thus not significant (Dhat1 = 0.872; CI = 0.871–0.873; *U* ^2^ = 0.03; critical value (cv) = 0.187; i.e., same probability distribution).

**FIGURE 5:**
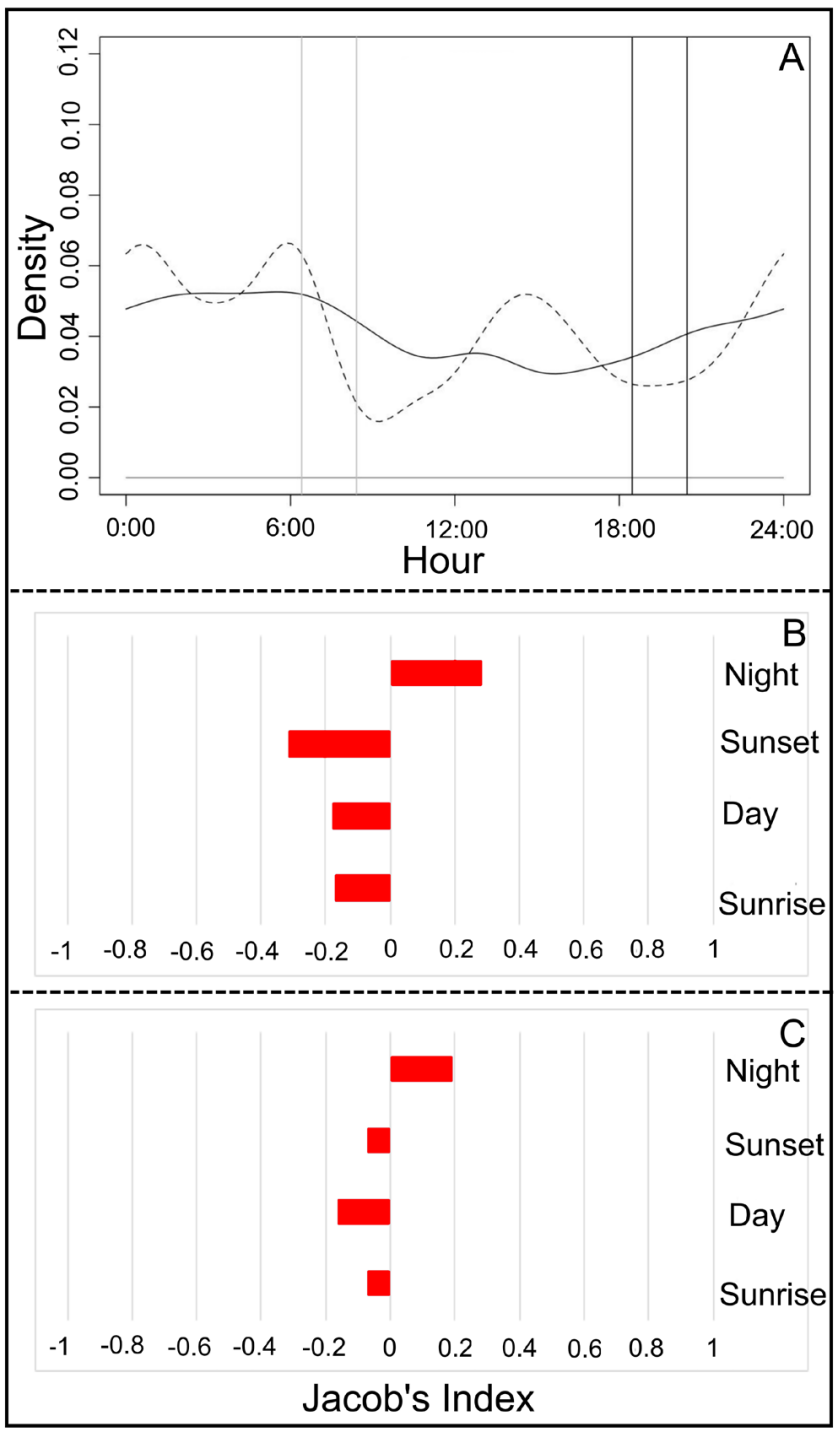
(A) Overlap of melanic (dashed line) and spotted (continuous line) morphotypes activity pattern. Gray and dark lines indicate sunrise and sunset phases, respectively. Jacob's index selection for melanic (B) and spotted (C) guigna's activity pattern.

We found the melanistic phenotype (*n* = 66) exhibited mostly nocturnal activity, with a much smaller peak occurring during daytime hours (*T* = 5.78, *p* = 0.084; Figure 5A). The JIS for the melanistic phenotype suggested a weak selection for nighttime hours (JIS = 0.28) and a weak avoidance for the other day phases (Figure 5B). Similarly, the spotted phenotype (*n* = 109) also exhibited a weak selection for night hours and minimal avoidance of other day phases (*T* = 3.95, *p* = 0.31; Figure 5C). Overlap in kernel activity densities between spotted and melanistic morphotypes was therefore also high (Dhat1 = 0.86, CI = 0.83–0.87) and not significant (*U* ^2^ = 0.04, cv = 0.187, i.e., same probability distribution).

**FIGURE 6:**
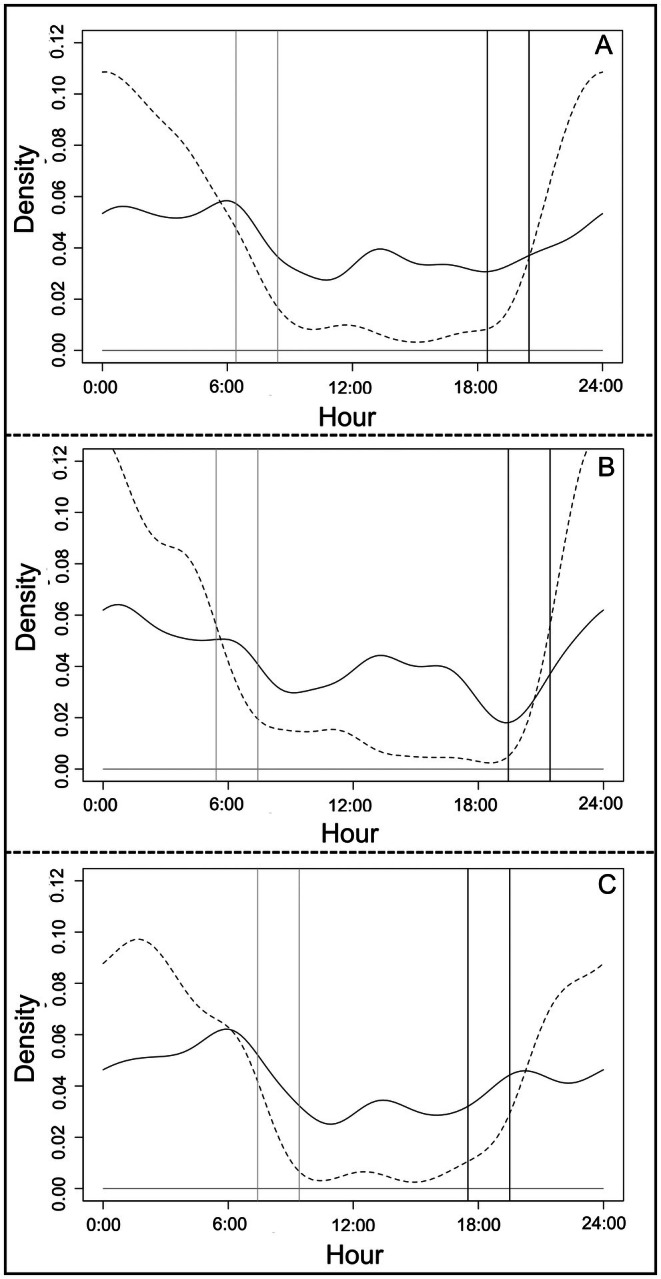
Guigna activity pattern (continuous line) overlapped with small mammals (dashed line) throughout the year (A), during the warm season (B) and during the cold season (C). Gray and dark lines indicate sunrise and sunset phases, respectively.

We found that guigna (*n* = 175) and small mammal (*n* = 181; *T* = 107.1, *p* = 0.001) activity patterns overlapped moderately throughout the year, and during each of the cold and warm seasons considered separately (Dhat1_year_ = 0.669, CI = 0.668–0.6687; Dhat1_cold_ = 0.6984, CI = 0.6983–0.7113; Dhat1_hot_ = 0.6462, CI = 0.6382–0.6682); kernel density curves mostly overlapped at night (Figure 6A–C). However, we did find a significant relationship between the curves (*U* ^2^_year_ = 1.18, cv = 0.187; *U* ^2^_cold_ = 0.62, cv = 0.187; *U* ^2^_hot_ = 0.56, cv = 0.187, i.e., differences between probability distribution).

**FIGURE 7:**
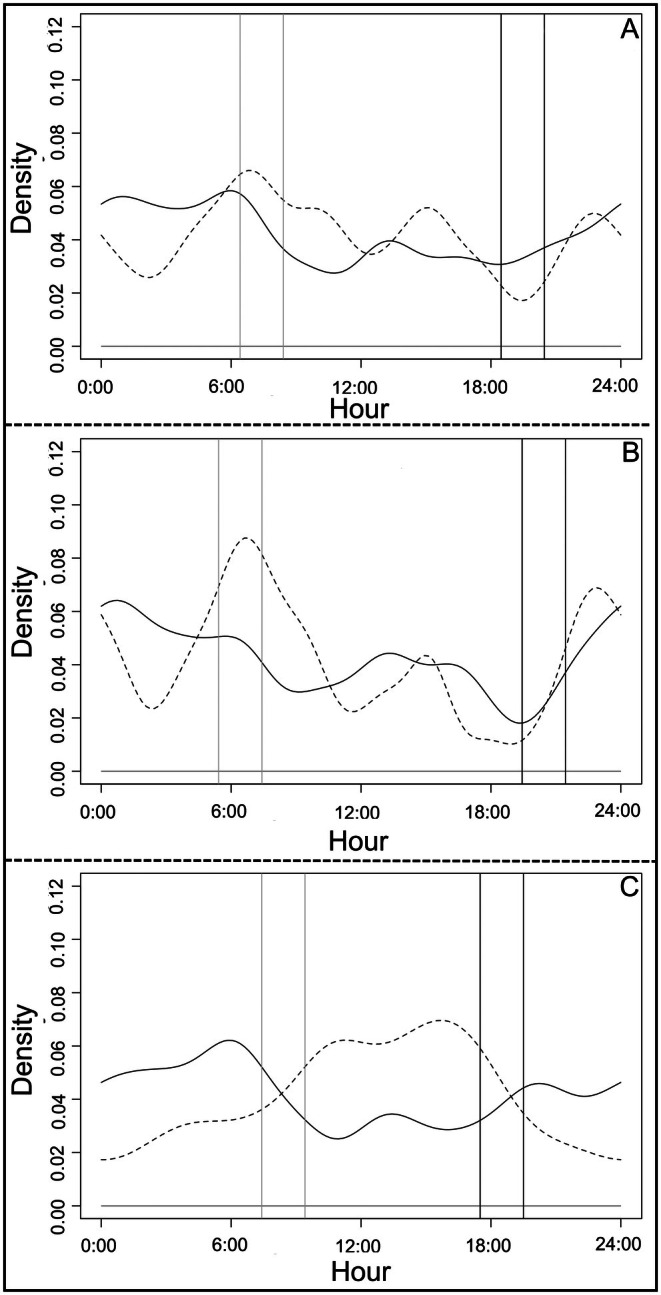
Guigna activity patterns (continuous line) overlapped with co‐predator activity patterns (dashed line) throughout the year (A), during warm (B) and cold seasons (C). Gray and dark lines indicate sunrise and sunset phases, respectively.

The activity patterns of guigna (*n* = 175) and co‐predators (*n* = 61; *T* = 2.64, *p* = 0.58) also overlapped. Overlap was relatively high throughout the year (Dhat1_year_ = 0.8491, CI = 0.8490–0.8792), and also during the warmer season (Dhat1_hot_ = 0.7960, CI = 0.761–0.7961). The cold season, in contrast, saw only moderate overlap (Dhat1_cold_ = 0.6878, CI = 0.670–0.6879). From the overlap in Kernel density curves, we found that detection events for guigna increased when those of co‐predators decreased (Figure 7A–C). We found non‐significant differences between these curves throughout the year and during the warmer season (*U* ^2^_year_ = 0.11, cv = 0.187; U^2^_hot_ = 0.07, cv = 0.187; i.e., same probability distribution); in contrast, we found a significant difference in the curves for the cold season (*U* ^2^_cold_ = 0.22, cv = 0.187; i.e., differences between probability distribution).

## 4 Discussion

**FIGURE 8:**
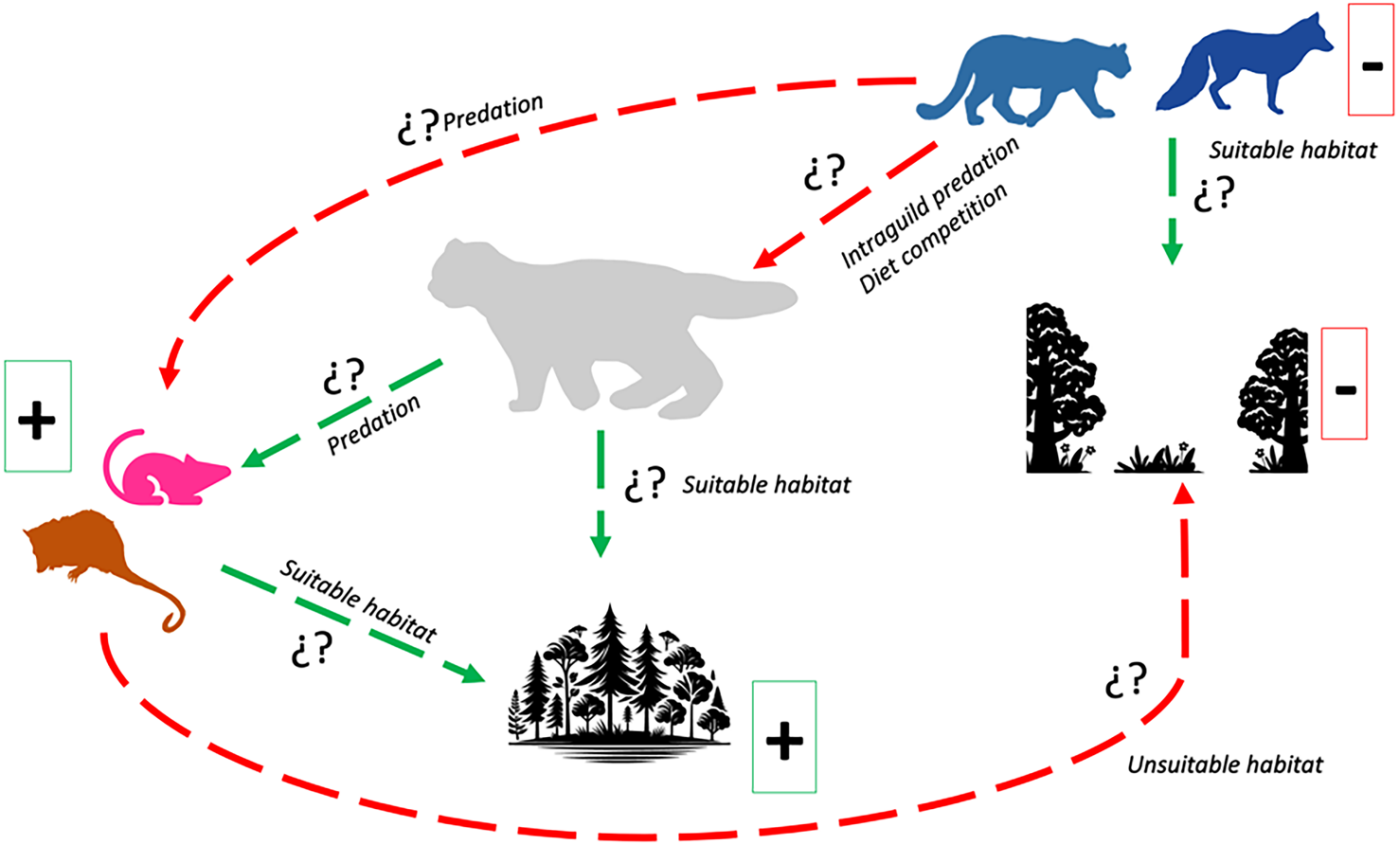
Graphical interpretation of the found and potential interactions between Grinnellian and Eltonian variables of guigna habitat use in the study area. The positive (“+”) and negative (“−”) signs indicate interactions we found in this study; dashed arrows indicate potential interactions. Red dashed arrows indicate negative interactions, whereas green dashed arrows indicate positive interactions.

In this study, we explored habitat use for the guigna in the context of an explicit theoretical framework that combined the use of Grinnellian and Eltonian variables (Figure 8). For those areas that were accessible, this allowed us to determine a portion of the Realized Niche (Soberón and Nakamura 2009) for this small felid. Because natural history traits can be temporarily variable in a given habitat (Schoener, 1974), we also examined these traits through time. In the end, this allowed us to develop the first multi‐annual information regarding the spatio‐temporal ecology of guigna in Argentina. However, we note that our results should be considered in their geographical context, bearing in mind that evaluation of broader habitat gradients and range‐wide patterns of selection may exist across the guigna's distribution and therefore remains to be analyzed.

Notably, our detection rate for guignas was among the highest recorded for the species (see Delibes‐Mateos et al. 2014; Gálvez et al. 2017; Zúñiga et al. 2025), and the highest for Argentina thus far (see Berrondo and Bravo 2022; Agostini et al. 2024). The sites we sampled in LANP mainly occurred in the western and southern areas of the PA, which is more of the “core” National Park (*sensu stricto*), where access by tourists is restricted (Figure 1; APN 2019). A previous study (see Berrondo and Bravo 2022) with camera traps in this PA had reported much lower guigna detection rates, which may have been due to their sampling of sites in the eastern portion of LANP, or the “National Reserve”, which is more accessible to humans (i.e., trails, roads, settlements, cattle).

We found that habitat use of the guigna was negatively affected by the “presence of co‐predators” (Figure 8). Competition between sympatric carnivorans can have different effects on different species, even triggering potential consequences that affect the conservation of local populations (Burrows et al. 1995). Caro and Stoner (2003) described five forms of competition between carnivorans, which can generate diet, habitat use, and behavior shifts on the “subordinate” species. In LANP, six terrestrial species of carnivorans are native (i.e., guigna, puma, Andean fox, *L. geoffroyi* , *C. chinga* , and *G. cuja* ). However, excluding the puma and Andean fox, the detection rate for the other carnivorans was either extremely low or zero. Thus, the negative effect identified on guigna habitat use can be assumed as coming from interactions (e.g., competition) with the two larger mentioned species, as both of them have larger body sizes (De Angelo et al. 2019; Pía et al. 2019; Figure 8). Aside from the density of these other carnivore species being low, it is also possible that camera traps were not the best way to survey for them. For example, we didn't specifically target otters with camera placement or use other more effective survey techniques. Moreover, although the trophic niches of the two larger carnivores may not overlap considerably with guigna due to morphological dissimilarities and size disparities (e.g., Morales and Giannini 2010; Bubadué et al. 2016), some food items may still be shared among them (e.g., small mammals; guigna: Figueroa et al. 2018; Andean fox: Osorio et al. 2020; puma: Guerisoli et al. 2021). Information on the diet for the guigna is actually lacking for Argentina, but small mammals are among the prey of both pumas (Guerisoli et al. 2021) and Andean foxes in other regions (Monteverde and Piudo 2011; Figure 8). Intraguild predation (see Polis et al. 1989; de Oliveira and Pereira 2014) could be another important interaction affecting the relationship with guignas (Figure 8). Although we are unaware of confirmed predation on guigna by pumas or Andean foxes, these predators are known to feed or kill other small felids (Zapata et al. 2005; de Oliveira and Pereira 2014; Osorio et al. 2020; Guerisoli et al. 2021; Figure 8). Remarkably, despite the fact that guigna and *L. geoffroyi* are believed to be sympatric in the study area (G. Bauer, pers. comm.), after 8024 night‐traps, we still hadn't confirmed the presence of the species. One previous hypothesis suggested that when both species co‐occur, the local guigna population could be at a low density because of competition with the larger *L. geoffroyi* (Lucherini et al. 2001; Agostini et al. 2024). However, we found no support for this potential interaction and actually, we found the lack of *L. geoffroyi* records to be remarkable. As they are not a threatened species however, we did not find this cause for concern; instead, we believe there is a need to further analyze the mechanisms of sympatry between these two felid species, and it is just possible that for *L. geoffroyi* , the habitat conditions in at LANP are sub‐optimal.

Vegetation categories also differentially affected guigna presence, with open sites containing less vegetation coverage (Mohr‐Bell et al. 2019) negatively affecting their presence. Previous studies from Chile found that guigna avoided open habitats (e.g., shrublands; Dunstone et al. 2002) where anthropogenic impacts might be greatest (e.g., dogs *Canis familiaris* , roads; Gálvez et al. 2013; Schüttler et al. 2017). However, in the areas we sampled in our study area, human disturbances can be considered low or null (APN 2019). Therefore, it is possible the negative effect of open areas may have to do with exposure to co‐predators, and thus the potential for intraguild predation (see above). Alternatively, this effect may have to do with the lower availability of guigna prey (e.g., Figueroa et al. 2018; Figure 8). Consistent with the findings of other studies, we found the vegetation category “MFL” positively affected guigna presence (Dunstone et al. 2002; Acosta‐Jammet and Simonetti, 2004; Gálvez et al. 2013). Dunstone et al. (2002) hypothesized that the preference by guigna for thick forests could be linked to increased prey availability. However, information on prey availability for the guigna is lacking in Argentina. Forests with high levels of heterogeneity (like “MFL”; see Mohr‐Bell et al. 2019) could also be affecting guigna's prey search efficiency and its survival (Figure 8), which is consistent with the idea that the guigna is a specialist of southern temperate forests of a certain age. Variation in structural and nonstructural forest features (*sensu* Mysterud and Østbye 1999) could provide greater opportunities for the guigna to more fully satisfy its natural history needs. Among these needs for example, may be more specific hunting and resting/hiding cover, or the presence of more den sites for reproduction, such as tree hollows as in the case of several small felid species. Indeed, the guigna's breeding (e.g., Novoa et al. 2025), sheltering (e.g., Vergara et al. 2024), and predation (e.g., Altamirano et al. 2013) behavior have all been previously recorded from forested environments (Figure 8).

The “presence of potential prey” also affected guigna presence. Here, we included only small mammals *sensu lato* as potential food resources (Figure 8), due to the fact that this group was reported as most consumed in other areas (e.g., Figueroa et al. 2018). Given that taxonomic resolution to the species level was not possible for most small mammal species we detected via camera traps, a more granular examination of species‐specific interactions between these mammals and guigna could not be performed. However, carnivoran distribution is usually strongly shaped by, among other variables, the distribution, presence, and abundance of prey, and then any shifts in prey populations (Fuller and Sievert 2001). One study of a small felid from Asia even suggested that an understanding of prey distribution can better help identify new areas for future surveys (Greenspan and Giordano 2021). Future studies could focus on the inclusion of species‐specific prey abundance indexes, which might better demonstrate how each affects guigna presence.

As expected, guigna displayed increased activity during the night. Previous studies from Chile had made similar conclusions, that is, the species was identified as mainly nocturnal (Delibes‐Mateos et al. 2014; Gálvez et al. 2021; Zúñiga et al. 2025). Seasonal activity patterns overlapped significantly, consistent with the results found in Chile (Galuppo Gaete 2014). This is despite the lack of anthropogenic disturbances on the landscape that might impact the guigna. We therefore found no evidence guigna avoided activity during the day due to anthropogenic activity, as it has been suggested by other authors (e.g., Gálvez et al. 2021; Zúñiga et al. 2025).

The melanistic guigna phenotype, caused by a recessive gene mutation (Schneider et al. 2015), only exhibited a weak preference for nocturnal activity. Hernández et al. (2015) came to similar conclusions in Chile, but the overlap between spotted and melanistic activity patterns was less than what we found here. They suggested that melanistic individuals might be enhancing their crypticism or camouflage in the nocturnal hunting of small mammals on the forest floor (Hernández et al. 2015). Future efforts might explore the relationship between these phenotypes further and over longer periods, as perhaps the answers may lie in longer‐term studies of the species.

Guigna and small mammals exhibited moderate overlap in their Kernel activity density curves, mainly at night. In Chile, previous studies also came to similar conclusions, but with higher overlap values (~> 0.7; Delibes‐Mateos et al. 2014; Gálvez et al. 2021; Zúñiga et al. 2025). Although our data suggests that guignas are active during hours when small mammals are not, they could still be using small mammals as prey as other studies have found (Figueroa et al. 2018 but also Delibes‐Mateos et al. 2014; Gálvez et al. 2021; Zúñiga et al. 2025). Perhaps guignas also employ strategies where they pursue small mammals in their resting and denning cover, as other small felids might (e.g., Pallas's cats *Otocolobus manul* and pikas *Ochotona* spp.; Greenspan and Giordano 2021). However, guignas could also be relying on other prey as well, which are mainly active during the day (e.g., birds; Zúñiga et al. 2025), and/or avoiding co‐predators during times of less activity. Future studies on the comparative feeding ecology of these species could help confirm or reject these potential hypotheses.

Finally, information on guigna activity patterns and that of co‐predators or competitors is scarce, and more investigations are needed to better illuminate these relationships across the guigna's range. In Chile, a prior study found a high overlap between guigna and the invasive domestic cat *Felis catus* , suggesting that guignas have not changed their behavior, despite the potential presence of interference competition (Gálvez et al. 2021). For our study however, we found overlap with much larger carnivorans, suggesting the potential for other agonistic interactions with species of similar body size (e.g., intraguild predation, resource competition; de Oliveira and Pereira 2014). Guignas could also be shifting their behavior in order to avoid Andean fox and pumas during the hotter seasons, and other differences we didn't detect between years. Temporal segregation or partitioning is a strategy used by other species to facilitate coexistence, and has been recorded in other regions for several carnivorans (e.g., Karanth et al. 2017; Brodie et al. 2018; Finnegan et al. 2021; Widodo et al. 2022; Krag et al. 2023; Havmøller, Wahyudi, et al. 2024; Havmøller, Parsons, et al. 2024). The overlap we found between guigna and co‐predators during the cold season could be due to a scarcity of resources, especially since small‐mammals appeared less abundant in this period (Garcia et al. 2013). Considering that the “presence of co‐predators” negatively affected guigna habitat use, future research might focus on spatio‐temporal segregation between this small felid and its co‐predators.

Here, we found a remarkable number of records for a little‐known species, showing positive associations with thick forests and potential prey, and negative associations with open environments and presence of co‐predators. We depicted guigna as a primarily nocturnal carnivoran, with shifting overlap patterns with co‐predators according to seasons. The Valdivian Temperate Forests inhabited by the guigna are a scarcely studied biodiversity hotspot, particularly in Argentina. The information generated in this study can be used for management plans in National Parks of Argentina where guigna has been detected (i.e., LANP, Lago Puelo National Park, Nahuel Huapi National Park; SIB 2026). Previous studies on the species are extremely limited in this country, making our results highly innovative, and highlighting the strong need for future research.

## 5 Possible Caveats in This Study

We can identify potential limitations in this study, mainly associated with the CTs placement and the prey detection and identification. First, the placement of the CTs has been somewhat limited by the availability of accessible grounds (see Figure 1 and Supporting Information S1). The western parts of LANP are highly inaccessible, and climatic conditions mostly complicate the field‐works (Figure 1; Guerisoli et al. 2025). Even more, in these remote areas, the thick forests with dense understories and the elevated slopes make it impossible to reach certain areas by foot. Thus, a stratified sampling design was not possible, and the selection of the sampling sites was limited to keep a minimal distance between CTs in order to avoid spatial autocorrelation.

Second, the “potential preys” variable was included in the analyses as a “categorical predictor” since, on one hand, the CTs were placed to maximize the capture of guignas (i.e., cameras set between 20 to 60 cm height; Sereno‐Cadierno et al. 2026), and, on the other hand, the impossibility to reach species‐level identification from photos (please refer to Material and Methods of this research for more details). Future research could better approach this by combining CTs with small‐mammal field surveys, in order to reach an availability measure of small mammals' diversity. However, as this is an endemic area for the Andes type of Hantavirus (Lázaro et al. 2007), field‐works with small mammals need to be made with extreme care and precautionary measures.

Finally, the chaotic and precarious economic situation in Argentina, with deprecated salaries and lack of financial support (Lambertucci et al. 2023), makes the development of scientific studies highly challenging. Although the buying of equipment was possible due to a Rufford Small Grant (The Rufford Foundation), finished in 2021, on the majority of occasions fieldwork was only possible by using personal salaries and vehicles. Even more, for 2026 the budget for science in Argentina will be at its historical lowest (Grupo 2025), making scientific research completed under this lamentable situation emotionally and logistically exhausting.

## Author Contributions

**M. M. Guerisoli:** conceptualization (equal), data curation (equal), formal analysis (equal), funding acquisition (equal), investigation (equal), methodology (equal), project administration (equal), resources (equal), software (equal), supervision (equal), validation (equal), visualization (equal), writing – original draft (equal), writing – review and editing (equal). **G. Bauer:** methodology (equal), writing – original draft (supporting). **S. Sarra Pistone:** data curation (equal), methodology (equal), writing – original draft (equal). **E. Buffa:** data curation (equal), writing – review and editing (equal). **C. Bonaglia:** data curation (equal), writing – review and editing (equal). **A. J. Giordano:** conceptualization (equal), funding acquisition (equal), methodology (equal), project administration (equal), resources (equal), writing – review and editing (equal). **M. I. Schiaffini:** conceptualization (equal), data curation (equal), formal analysis (equal), funding acquisition (equal), investigation (equal), methodology (equal), project administration (equal), resources (equal), software (equal), supervision (equal), validation (equal), visualization (equal), writing – original draft (equal), writing – review and editing (equal).

## Funding

This work was supported by the Rufford Foundation (34575‐1).

## Conflicts of Interest

The authors declare no conflicts of interest.

## Supporting information


**Data S1:** Challenging approach to the coast in Los Alerces National Park to install/control camera traps.

**Data S2:** Residuals plot of the global model of guigna habitat use using the “simulateResiduals” function in DHARMa package (Hartig 2016).

**Data S3:** List of models obtained from the “dredge” function. Models have been ordered from the lowest AIC. AIC, Akaike Information Criteria; Co‐pred, presence/absence of co‐predators; df, degrees of freedom; Int, intercept; MFL, mixed forested land; NA, not included; NDVI, Normalized Difference Vegetation Index; OWF, Other Woody Formations; Potential prey, presence/absence of potential prey; SHI, Shannon Diversity Index. Null model is in bold.

## Data Availability

Presence data of the species is available in the manuscript (see Figure 1). Specific presence data (i.e., capture rate per site) of the species is not available since is a endangered species in Argentina and threatened by illegal hunting due to poultry predation.
