# Supplementary material for: Integrating Niche Dimensions to Advance the Ecological Study of the Americas' Smallest Cat: The Guigna in Argentine Patagonia
Source: Ecol Evol. 2026 May 30;16(6):e73704. doi: 10.1002/ece3.73704 (PMC13239121; doi:10.1002/ece3.73704)
Supplement: Supplementary file 1 — Data S1: Challenging approach to the coast in Los Alerces National Park to install/control camera traps. [file ECE3-16-e73704-s001.zip › Media/Transcription text.docx]

Challenging approach to the coast in Los Alerces National Park to install/control camera traps.
