# Supplementary material for: Integrating Niche Dimensions to Advance the Ecological Study of the Americas' Smallest Cat: The Guigna in Argentine Patagonia
Source: Ecol Evol. 2026 May 30;16(6):e73704. doi: 10.1002/ece3.73704 (PMC13239121; doi:10.1002/ece3.73704)
Supplement: Supplementary file 2 — Data S2: Residuals plot of the global model of guigna habitat use using the “simulateResiduals” function in DHARMa package (Hartig 2016). [file ECE3-16-e73704-s002.docx]

**Supplementary Material 2**. Residuals plot of the global model of guigna habitat use using the ‘simulateResiduals’ function in DHARMa package (Hartig, 2016).


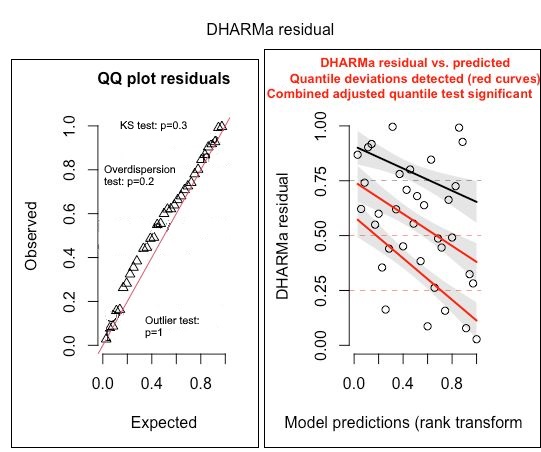


Hartig, F. (2016). DHARMa: residual diagnostics for hierarchical (multi-level/mixed) regression models. CRAN: contributed packages.
