# Supplementary material for: Integrating Niche Dimensions to Advance the Ecological Study of the Americas' Smallest Cat: The Guigna in Argentine Patagonia
Source: Ecol Evol. 2026 May 30;16(6):e73704. doi: 10.1002/ece3.73704 (PMC13239121; doi:10.1002/ece3.73704)
Supplement: Supplementary file 3 — Data S3: List of models obtained from the “dredge” function. Models have been ordered from the lowest AIC. AIC, Akaike Information Criteria; Co‐pred, presence/absence of co‐predators; df, degrees of freedom; Int, intercept; MFL, mixed forested land; NA, not included; NDVI, Normalized Difference Vegetation Index; OWF, Other Woody Formations; Potential prey, presence/absence of potential prey; SHI, Shannon Diversity Index. Null model is in bold. [file ECE3-16-e73704-s003.docx]

From manuscript “Integrating niche dimensions to advance the ecological study of the Americas’ smallest cat: the guigna in Argentine Patagonia”. Guerisoli M.. Bauer G.. Sarra Pistone S.. Buffa E.. Bonaglia C.. Giordano A. J.. Schiaffini. M.

**Supplementary Material 3.** List of models obtained from the ‘dredge’ function. Models have been ordered from the lowest AIC. Int: Intercept; “Co-pred”: Presence/absence of co-predators; “NDVI”: Normalized Difference Vegetation Index; “OWF”: Other Woody Formations; “Potential prey”: Presence/absence of potential prey; “SHI”: Shannon Diversity Index; “MFL”: Mixed Forested Land; df: degrees of freedom; AIC: Akaike Information Criteria; NA: Not Included. Null model is in bold.

| Int. | Co-pred | NDVI | OWF | Potential prey | SHI | MFL | offset(log(sampling effort)) | df | logLik | AIC | Delta | Weight |
| --- | --- | --- | --- | --- | --- | --- | --- | --- | --- | --- | --- | --- |
| -6.4791856 | + | NA | -7.5192172 | + | NA | 0.40312042 | + | 6 | -69.76807 | 151.53614 | 0 | 0.25079901 |
| -6.5646973 | + | 0.13258979 | -8.0127591 | + | NA | 0.36942627 | + | 7 | -69.630486 | 153.260971 | 1.72483116 | 0.10587258 |
| -6.4695767 | + | NA | -7.4622518 | + | -0.0169859 | 0.40665591 | + | 7 | -69.765798 | 153.531597 | 1.99545657 | 0.09247364 |
| -6.2021391 | + | NA | -8.3963658 | NA | NA | 0.39617369 | + | 5 | -71.873161 | 153.746322 | 2.21018181 | 0.08305981 |
| -6.4451569 | + | NA | -7.7814339 | + | NA | NA | + | 5 | -72.028698 | 154.057396 | 2.52125592 | 0.07109549 |
| -6.5823213 | + | 0.30105387 | -8.682142 | + | NA | NA | + | 6 | -71.32949 | 154.658979 | 3.12283908 | 0.05262716 |
| -6.6076076 | + | 0.15263173 | -8.2517723 | + | 0.04608664 | 0.35526414 | + | 8 | -69.616546 | 155.233091 | 3.69695123 | 0.03949509 |
| -6.2620282 | + | 0.13184373 | -8.8139452 | NA | NA | 0.35997626 | + | 6 | -71.754749 | 155.509497 | 3.97335719 | 0.03439714 |
| -6.0506457 | + | NA | -7.9977835 | NA | NA | NA | + | 4 | -73.795039 | 155.590078 | 4.05393759 | 0.03303882 |
| -6.1807462 | + | NA | -8.2493374 | NA | -0.0458792 | 0.4069217 | + | 6 | -71.857784 | 155.715568 | 4.1794276 | 0.03102949 |
| -6.5104989 | + | NA | -8.1604338 | + | 0.13473574 | NA | + | 6 | -71.897926 | 155.795852 | 4.25971175 | 0.02980857 |
| -6.7794246 | + | 0.36522671 | -9.6657713 | + | 0.22618072 | NA | + | 7 | -70.969458 | 155.938916 | 4.40277631 | 0.02775077 |
| -6.1418711 | + | 0.31081001 | -8.8335555 | NA | NA | NA | + | 5 | -73.126264 | 156.252527 | 4.71638729 | 0.02372331 |
| -6.102979 | + | NA | -8.3378207 | NA | 0.11274944 | NA | + | 5 | -73.707406 | 157.414812 | 5.87867149 | 0.01326748 |
| -6.2644316 | + | 0.1332786 | -8.8300698 | NA | 0.00348106 | 0.35881855 | + | 7 | -71.754672 | 157.509345 | 5.97320471 | 0.01265496 |
| -6.2615834 | + | 0.35048134 | -9.5570922 | NA | 0.17413436 | NA | + | 6 | -72.916314 | 157.832628 | 6.29648826 | 0.01076616 |
| -6.7111006 | NA | NA | -6.8705029 | + | NA | 0.286755 | + | 5 | -74.288889 | 158.577778 | 7.04163787 | 0.00741743 |
| -6.523726 | NA | NA | -6.843054 | + | NA | NA | + | 4 | -75.318096 | 158.636192 | 7.10005224 | 0.00720392 |
| -6.5430519 | NA | NA | -6.0530963 | + | -0.3388194 | 0.38666974 | + | 6 | -73.420048 | 158.840096 | 7.30395584 | 0.00650567 |
| -6.120266 | NA | NA | -6.9745334 | NA | NA | NA | + | 3 | -76.485412 | 158.970824 | 7.43468365 | 0.00609403 |
| -6.5504159 | NA | -0.2911322 | -6.1559147 | + | NA | 0.38127447 | + | 6 | -73.609388 | 159.218777 | 7.68263645 | 0.00538347 |
| -6.3831613 | NA | -0.2865434 | -5.2875397 | + | -0.3507357 | 0.49151794 | + | 7 | -72.665754 | 159.331508 | 7.79536795 | 0.00508842 |
| -6.2851122 | NA | NA | -7.2801545 | NA | NA | 0.21909743 | + | 4 | -75.908133 | 159.816267 | 8.28012674 | 0.00399318 |
| -1.1269817 | NA | NA | -4.8912326 | + | NA | 0.33025477 | NA | 5 | -74.940108 | 159.880215 | 8.34407508 | 0.00386752 |
| -0.9693967 | + | NA | -5.1068716 | + | NA | 0.36419299 | NA | 6 | -74.056407 | 160.112814 | 8.57667423 | 0.0034429 |
| -6.1840103 | NA | NA | -6.5900391 | NA | -0.3537279 | 0.34752191 | + | 5 | -75.060686 | 160.121372 | 8.58523176 | 0.0034282 |
| -6.4442681 | NA | NA | -6.5040984 | + | -0.1679472 | NA | + | 5 | -75.111005 | 160.222011 | 8.68587062 | 0.00325997 |
| -6.463622 | NA | -0.1144611 | -6.6168503 | + | NA | NA | + | 5 | -75.206221 | 160.412443 | 8.87630253 | 0.00296388 |
| -6.187253 | NA | -0.310572 | -6.7155239 | NA | NA | 0.34074011 | + | 5 | -75.219666 | 160.439333 | 8.90319245 | 0.0029243 |
| -6.039404 | NA | NA | -6.597281 | NA | -0.1761106 | NA | + | 4 | -76.259215 | 160.518431 | 8.98229092 | 0.00281091 |
| -6.0712205 | NA | -0.1263574 | -6.7581644 | NA | NA | NA | + | 4 | -76.351477 | 160.702953 | 9.16681315 | 0.00256317 |
| -6.0727475 | NA | -0.2919756 | -6.0069424 | NA | -0.3518413 | 0.46703735 | + | 6 | -74.386086 | 160.772171 | 9.23603111 | 0.00247598 |
| -1.0236675 | NA | NA | -5.0873894 | + | NA | NA | NA | 4 | -76.447422 | 160.894845 | 9.35870477 | 0.00232868 |
| -1.0558712 | NA | NA | -4.5451071 | + | -0.1921835 | 0.38168959 | NA | 6 | -74.645709 | 161.291419 | 9.75527891 | 0.00190983 |
| -1.0909952 | NA | -0.1251993 | -4.6527024 | + | NA | 0.3673448 | NA | 6 | -74.78867 | 161.577341 | 10.0412004 | 0.00165542 |
| -0.9347195 | + | NA | -5.3778788 | + | NA | NA | NA | 5 | -75.885374 | 161.770748 | 10.2346075 | 0.00150283 |
| -0.9529847 | + | NA | -4.9274195 | + | -0.0859014 | 0.38501965 | NA | 7 | -74.002433 | 162.004867 | 10.4687266 | 0.00133681 |
| -6.4095178 | NA | -0.0899313 | -6.3634151 | + | -0.1530551 | NA | + | 6 | -75.040656 | 162.081313 | 10.5451728 | 0.00128668 |
| -0.9731324 | + | 0.04241897 | -5.2141473 | + | NA | 0.35386612 | NA | 7 | -74.0429 | 162.0858 | 10.5496596 | 0.0012838 |
| -6.0179812 | NA | -0.0898033 | -6.5020701 | NA | -0.1529743 | NA | + | 5 | -76.193435 | 162.386871 | 10.8507306 | 0.00110438 |
| -1.0125861 | NA | NA | -5.0402204 | + | -0.0320548 | NA | NA | 5 | -76.439315 | 162.87863 | 11.3424903 | 0.00086365 |
| -1.0289149 | NA | 0.01917095 | -5.114948 | + | NA | NA | NA | 5 | -76.443714 | 162.887429 | 11.3512886 | 0.00085985 |
| -1.0180437 | NA | -0.1421711 | -4.2621475 | + | -0.2081376 | 0.43028086 | NA | 7 | -74.444445 | 162.888891 | 11.3527506 | 0.00085923 |
| -0.9507887 | + | 0.19345446 | -5.8000016 | + | NA | NA | NA | 6 | -75.59478 | 163.18956 | 11.6534198 | 0.0007393 |
| -0.950486 | + | NA | -5.5278853 | + | 0.07877704 | NA | NA | 6 | -75.841499 | 163.682998 | 12.1468575 | 0.00057766 |
| -0.9545178 | + | 0.01263622 | -4.9678204 | + | -0.0811449 | 0.38075753 | NA | 8 | -74.001383 | 164.002767 | 12.466627 | 0.0004923 |
| -1.0175544 | NA | 0.02047132 | -5.0676152 | + | -0.0330719 | NA | NA | 6 | -76.435081 | 164.870161 | 13.3340214 | 0.00031907 |
| -0.9955036 | + | 0.22488603 | -6.1683709 | + | 0.13188312 | NA | NA | 7 | -75.47566 | 164.951321 | 13.4151806 | 0.00030638 |
| -4.7413776 | NA | -0.4782353 | NA | + | -0.549898 | 0.71117508 | + | 6 | -76.822913 | 165.645826 | 14.1096862 | 0.00021649 |
| -4.4788008 | + | -0.3687788 | NA | + | -0.4462278 | 0.70147573 | + | 7 | -76.404285 | 166.808571 | 15.2724306 | 0.00012105 |
| -4.2064233 | + | NA | NA | + | -0.3710483 | 0.59941508 | + | 6 | -77.499392 | 166.998785 | 15.4626447 | 0.00011007 |
| -3.989029 | + | NA | NA | + | NA | 0.54238593 | + | 5 | -78.515597 | 167.031194 | 15.4950544 | 0.0001083 |
| -4.1330447 | NA | -0.5109114 | NA | NA | -0.5813569 | 0.72539954 | + | 5 | -78.775591 | 167.551181 | 16.015041 | 8.35E-05 |
| -4.176769 | + | -0.2996445 | NA | + | NA | 0.60854629 | + | 6 | -77.894424 | 167.788849 | 16.2527087 | 7.41E-05 |
| -4.6288111 | NA | NA | NA | + | -0.5925525 | 0.56268991 | + | 5 | -79.152531 | 168.305063 | 16.7689229 | 5.73E-05 |
| -3.8689357 | + | -0.397579 | NA | NA | -0.490456 | 0.73194535 | + | 6 | -78.427053 | 168.854106 | 17.3179657 | 4.35E-05 |
| -4.6041647 | NA | -0.5773327 | NA | + | NA | 0.58908529 | + | 5 | -79.435914 | 168.871827 | 17.335687 | 4.31E-05 |
| -3.584045 | + | NA | NA | NA | -0.4261687 | 0.60672516 | + | 5 | -79.485957 | 168.971915 | 17.4357749 | 4.10E-05 |
| -3.2880373 | + | NA | NA | NA | NA | 0.5347799 | + | 4 | -80.809015 | 169.618029 | 18.0818894 | 2.97E-05 |
| -4.0319395 | NA | NA | NA | NA | -0.6468019 | 0.52265315 | + | 4 | -80.963715 | 169.92743 | 18.3912894 | 2.55E-05 |
| -0.1861449 | NA | NA | -5.5490703 | NA | NA | NA | NA | 3 | -82.005244 | 170.010488 | 18.4743476 | 2.44E-05 |
| 0.20055868 | NA | -0.3610912 | NA | + | -0.4713965 | 0.67371752 | NA | 6 | -79.076686 | 170.153373 | 18.6172328 | 2.27E-05 |
| -3.5144386 | + | -0.3368758 | NA | NA | NA | 0.61200785 | + | 5 | -80.183582 | 170.367165 | 18.8310245 | 2.04E-05 |
| -3.9700465 | NA | -0.6377871 | NA | NA | NA | 0.56255417 | + | 4 | -81.399745 | 170.799489 | 19.2633494 | 1.65E-05 |
| -0.3350987 | NA | NA | -5.8002603 | NA | NA | 0.24809358 | NA | 4 | -81.422367 | 170.844735 | 19.3085947 | 1.61E-05 |
| 0.28897642 | NA | NA | NA | + | -0.4899167 | 0.58389594 | NA | 5 | -80.496167 | 170.992335 | 19.4561944 | 1.49E-05 |
| -0.3049066 | + | NA | -6.5372072 | NA | NA | 0.37269025 | NA | 5 | -80.49953 | 170.99906 | 19.46292 | 1.49E-05 |
| -0.1306422 | + | NA | -5.9481664 | NA | NA | NA | NA | 4 | -81.633876 | 171.267753 | 19.7316127 | 1.30E-05 |
| -3.8578864 | + | NA | NA | + | NA | NA | + | 4 | -81.651508 | 171.303017 | 19.7668766 | 1.28E-05 |
| -0.2799088 | NA | NA | -5.3104779 | NA | -0.3019815 | 0.38163705 | NA | 5 | -80.920425 | 171.84085 | 20.3047102 | 9.78E-06 |
| -0.1529803 | NA | NA | -5.3837102 | NA | -0.0894037 | NA | NA | 4 | -81.954464 | 171.908929 | 20.3727885 | 9.45E-06 |
| -0.1924531 | NA | 0.03034446 | -5.5820256 | NA | NA | NA | NA | 4 | -81.998225 | 171.996449 | 20.4603092 | 9.05E-06 |
| 0.13163754 | + | -0.3910663 | NA | + | -0.4941896 | 0.6765789 | NA | 7 | -79.046947 | 172.093893 | 20.5577531 | 8.62E-06 |
| 0.24245468 | NA | -0.3962839 | NA | + | NA | 0.57844481 | NA | 5 | -81.100613 | 172.201226 | 20.6650856 | 8.17E-06 |
| -0.1441539 | + | 0.30722628 | -6.6466533 | NA | NA | NA | NA | 5 | -81.192641 | 172.385282 | 20.849142 | 7.45E-06 |
| -4.5378605 | NA | NA | NA | + | NA | 0.42874946 | + | 4 | -82.234516 | 172.469032 | 20.9328917 | 7.14E-06 |
| -3.9705984 | + | NA | NA | + | -0.2501336 | NA | + | 5 | -81.278353 | 172.556707 | 21.0205666 | 6.84E-06 |
| 0.4280642 | + | NA | NA | + | -0.4312177 | 0.59310615 | NA | 6 | -80.301552 | 172.603104 | 21.0669642 | 6.68E-06 |
| -0.3196366 | NA | -0.1109921 | -5.6667122 | NA | NA | 0.29215853 | NA | 5 | -81.34166 | 172.68332 | 21.1471796 | 6.42E-06 |
| -3.3685163 | + | NA | NA | NA | NA | NA | + | 3 | -83.354885 | 172.70977 | 21.1736298 | 6.33E-06 |
| -0.3369734 | + | 0.16851739 | -6.9821209 | NA | NA | 0.33664174 | NA | 6 | -80.365291 | 172.730582 | 21.1944415 | 6.27E-06 |
| -0.2643982 | + | NA | -6.1196199 | NA | -0.1519176 | 0.42010208 | NA | 6 | -80.389067 | 172.778135 | 21.2419947 | 6.12E-06 |
| -4.3115941 | NA | NA | NA | + | -0.467325 | NA | + | 4 | -82.421494 | 172.842988 | 21.3068478 | 5.92E-06 |
| -3.884133 | NA | NA | NA | NA | -0.4920097 | NA | + | 3 | -83.441692 | 172.883385 | 21.3472447 | 5.81E-06 |
| 0.31987517 | NA | NA | NA | + | NA | 0.48816996 | NA | 4 | -82.619918 | 173.239836 | 21.7036963 | 4.86E-06 |
| -0.1454774 | + | NA | -6.0800335 | NA | 0.04548324 | NA | NA | 5 | -81.623846 | 173.247693 | 21.7115526 | 4.84E-06 |
| -3.8997896 | + | -0.0690999 | NA | + | NA | NA | + | 5 | -81.625274 | 173.250548 | 21.7144076 | 4.83E-06 |
| 0.61418674 | + | NA | NA | + | NA | 0.52107136 | NA | 5 | -81.735989 | 173.471977 | 21.9358372 | 4.33E-06 |
| -3.5586121 | + | NA | NA | NA | -0.3181261 | NA | + | 4 | -82.748406 | 173.496813 | 21.9606727 | 4.27E-06 |
| -0.2582731 | NA | -0.1129628 | -5.1526569 | NA | -0.3060549 | 0.43044553 | NA | 6 | -80.833608 | 173.667216 | 22.1310764 | 3.92E-06 |
| -4.3560058 | NA | -0.267187 | NA | + | -0.4033513 | NA | + | 5 | -81.856404 | 173.712808 | 22.1766678 | 3.83E-06 |
| -0.1582083 | NA | 0.05287044 | -5.4137916 | NA | -0.102892 | NA | NA | 5 | -81.934136 | 173.868272 | 22.3321322 | 3.55E-06 |
| -4.306664 | NA | -0.3791504 | NA | + | NA | NA | + | 4 | -82.943323 | 173.886647 | 22.3505065 | 3.52E-06 |
| 0.38413573 | + | -0.3245105 | NA | + | NA | 0.57861502 | NA | 6 | -80.96843 | 173.936861 | 22.4007206 | 3.43E-06 |
| -3.8973014 | NA | -0.2339984 | NA | NA | -0.4180676 | NA | + | 4 | -83.03252 | 174.06504 | 22.5288997 | 3.22E-06 |
| -4.2744397 | NA | NA | NA | + | NA | NA | + | 3 | -84.047227 | 174.094454 | 22.5583139 | 3.17E-06 |
| -0.2031641 | + | 0.33988169 | -7.1302138 | NA | 0.11904015 | NA | NA | 6 | -81.125177 | 174.250355 | 22.7142149 | 2.93E-06 |
| -3.8349739 | NA | -0.3783449 | NA | NA | NA | NA | + | 3 | -84.165381 | 174.330762 | 22.7946222 | 2.82E-06 |
| -4.0353876 | + | -0.0971743 | NA | + | -0.2617359 | NA | + | 6 | -81.222373 | 174.444747 | 22.9086068 | 2.66E-06 |
| -3.7907844 | NA | NA | NA | NA | NA | NA | + | 2 | -85.252401 | 174.504801 | 22.9686613 | 2.58E-06 |
| -3.4227598 | + | -0.0789644 | NA | NA | NA | NA | + | 4 | -83.325856 | 174.651712 | 23.1155716 | 2.40E-06 |
| -0.2989462 | + | 0.12455536 | -6.5841632 | NA | -0.0975261 | 0.37532118 | NA | 7 | -80.32817 | 174.65634 | 23.1202001 | 2.39E-06 |
| -3.8408977 | NA | NA | NA | NA | NA | 0.29768205 | + | 3 | -84.391121 | 174.782242 | 23.2461017 | 2.25E-06 |
| -3.6204599 | + | -0.0913398 | NA | NA | -0.3225947 | NA | + | 5 | -82.70373 | 175.40746 | 23.8713197 | 1.64E-06 |
| 0.55206858 | NA | NA | NA | + | NA | NA | NA | 3 | -85.22872 | 176.457441 | 24.9213007 | 9.72E-07 |
| 0.53612845 | NA | NA | NA | + | -0.365514 | NA | NA | 4 | -84.241906 | 176.483813 | 24.9476729 | 9.59E-07 |
| 0.76101565 | + | NA | NA | + | NA | NA | NA | 4 | -84.738249 | 177.476498 | 25.9403578 | 5.84E-07 |
| 0.51712595 | NA | -0.2370336 | NA | + | NA | NA | NA | 4 | -84.745903 | 177.491805 | 25.955665 | 5.80E-07 |
| 0.49786972 | NA | -0.2000586 | NA | + | -0.3454552 | NA | NA | 5 | -83.885831 | 177.771661 | 26.2355212 | 5.04E-07 |
| 0.63877385 | + | NA | NA | + | -0.3108134 | NA | NA | 5 | -84.141107 | 178.282214 | 26.7460742 | 3.90E-07 |
| 0.66423911 | + | -0.1513956 | NA | + | NA | NA | NA | 5 | -84.595404 | 179.190808 | 27.6546678 | 2.48E-07 |
| 0.50360389 | + | -0.1972408 | NA | + | -0.3430432 | NA | NA | 6 | -83.885632 | 179.771264 | 28.2351238 | 1.85E-07 |
| 1.3060343 | NA | -0.390151 | NA | NA | -0.6591135 | 0.80797158 | NA | 5 | -85.582361 | 181.164721 | 29.6285812 | 9.24E-08 |
| 1.37313726 | NA | NA | NA | NA | -0.6803539 | 0.63859988 | NA | 4 | -86.658586 | 181.317172 | 29.7810315 | 8.56E-08 |
| 1.54439058 | + | NA | NA | NA | -0.6036869 | 0.6834447 | NA | 5 | -86.509023 | 183.018045 | 31.4819052 | 3.66E-08 |
| 1.20830937 | + | -0.4323664 | NA | NA | -0.6918914 | 0.79975494 | NA | 6 | -85.550887 | 183.101773 | 31.565633 | 3.51E-08 |
| 1.53590216 | NA | NA | NA | NA | -0.4493082 | NA | NA | 3 | -89.270677 | 184.541354 | 33.0052137 | 1.71E-08 |
| 1.47934449 | NA | -0.4525186 | NA | NA | NA | 0.53441956 | NA | 4 | -88.400451 | 184.800902 | 33.2647622 | 1.50E-08 |
| **1.61513593** | **NA** | **NA** | **NA** | **NA** | **NA** | **NA** | **NA** | **2** | **-90.451758** | **184.903516** | **33.3673754** | **1.42E-08** |
| 1.9533798 | + | NA | NA | NA | NA | 0.53266587 | NA | 4 | -88.519529 | 185.039059 | 33.5029187 | 1.33E-08 |
| 1.55964802 | NA | NA | NA | NA | NA | 0.33088259 | NA | 3 | -89.629623 | 185.259246 | 33.7231056 | 1.19E-08 |
| 1.73165896 | + | -0.2955135 | NA | NA | NA | 0.58046409 | NA | 5 | -88.175895 | 186.351791 | 34.8156507 | 6.91E-09 |
| 1.80212226 | + | NA | NA | NA | NA | NA | NA | 3 | -90.19447 | 186.388941 | 34.8528008 | 6.78E-09 |
| 1.59759404 | NA | -0.19841 | NA | NA | NA | NA | NA | 3 | -90.197221 | 186.394443 | 34.8583026 | 6.76E-09 |
| 1.5327583 | NA | -0.0883715 | NA | NA | -0.4271833 | NA | NA | 4 | -89.219739 | 186.439478 | 34.9033374 | 6.61E-09 |
| 1.50797934 | + | NA | NA | NA | -0.464459 | NA | NA | 4 | -89.267076 | 186.534152 | 34.9980115 | 6.30E-09 |
| 1.71624693 | + | -0.1132175 | NA | NA | NA | NA | NA | 4 | -90.152715 | 188.305431 | 36.7692907 | 2.60E-09 |
| 1.38004396 | + | -0.1684233 | NA | NA | -0.4858369 | NA | NA | 5 | -89.15458 | 188.309161 | 36.7730208 | 2.60E-09 |
